# Seasonal Activity of Urban Bats Populations in Temperate Climate Zone—A Case Study from Southern Poland

**DOI:** 10.3390/ani11051474

**Published:** 2021-05-20

**Authors:** Joanna Kohyt, Ewa Pierzchała, Andrea Pereswiet-Soltan, Krzysztof Piksa

**Affiliations:** 1 Institute of Biology, Biotechnology and Environmental Protection, Faculty of Natural Sciences, University of Silesia in Katowice, Bankowa 9, 40-007 Katowice, Poland; 2 Doctoral School of Exact and Natural Sciences, Engineering and Technology, University of Silesia in Katowice, Bankowa 14, 40-007 Katowice, Poland; ewa.pierzchala@us.edu.pl; 3 Institute of Systematics and Evolution of Animals, Polish Academy of Sciences, Sławkowska 17, 31-016 Kraków, Poland; pereswiet_soltan@yahoo.it; 4 Department of Zoology, Institute of Biology, Pedagogical University of Krakow, Podchorążych 2, 30-084 Kraków, Poland; krzysztof.piksa@up.krakow.pl

**Keywords:** city, urban bats, Chiroptera, temperate zone, seasonal activity, municipal greenery, *Pipistrellus kuhlii*, *Hypsugo savii*

## Abstract

**Simple Summary:**

Urban green areas are essential for many animals inhabiting cities, including bats. They provide food and shelter, and also facilitate migration. Our aim was to identify bat species inhabiting Planty Park in Cracow and determine how their activity differed depending on the weather and season. We recorded bats’ calls on ultrasonic detectors in 2016 and 2017. In total, 2 of 10 observed species were new for this part of Poland: the Kuhl’s pipistrelle (*Pipistrellus kuhlii*) and the Savi’s pipistrelle (*Hypsugo savii*). We divided all species into groups of similar ecology for further analyses. Myotis bats were the least active group. Bats of genera *Nyctalus*, *Eptesicus* and *Vespertilio* were the most active in late summer, similarly to *Pipistrellus* and *Hypsugo*, although statistics did not back the outcome for the latter two genera. In spring and early summer, *Nyctalus*, *Eptesicus* and *Vespertilio* bats were more active during warmer nights, while in autumn, they preferred cloudless nights. Interestingly, *Pipistrellus* and *Hypsugo* bats decreased their activity at higher temperatures during summer. Our study will lead to a better understanding of bat ecology in urban areas and will contribute to setting urban landscape planning recommendations.

**Abstract:**

Municipal greenery can mitigate the negative impact of urbanization on biodiversity, including bats, by providing a migration corridor, food base and roosts. Our study aimed to evaluate the species composition and diversity, test the differences in activity between seasons, and identify the atmospheric conditions influencing the bats’ activity in the Planty Park (Cracow). Fieldworks were conducted in 2016 and 2017. We recorded 10 species, two new for this part of Poland: the Kuhl’s pipistrelle (*Pipistrellus kuhlii*) and the Savi’s pipistrelle (*Hypsugo savii*). Taxa were divided into three ecological guilds. *Myotis* group’s activity was insufficient to perform statistical analyses. The activity of *Nyctalus*, *Eptesicus* and *Vespertilio* group peaked in late summer. A similar insignificant trend was observed for *Pipistrellus* and *Hypsugo*. Temperature enhanced the activity of *Nyctalus*, *Eptesicus* and *Vespertilio* group in spring and early summer, while cloud cover suppressed their activity in autumn. Temperature also enhanced *Pipistrellus* and *Hypsugo* group activity in spring and autumn, but it suppressed their summer activity. Our study is one of the first to investigate temperate urban bats’ phenology and may serve as a preface for further research to introduce detailed urban landscape planning recommendations.

## 1. Introduction

Urbanization, understood as the process of urban development, is indicated as a threat to biodiversity, including bats [1,2,3]. This phenomenon entails natural habitats’ loss, fragmentation or disturbance. Urban environments differ from natural ones due to the constant presence of humans, characterized by increased levels of artificial light and noise, pollution, and changes in mesoclimatic conditions [1]. Notably, light and noise are often mentioned as important factors influencing bats’ activity and behavior in urban habitats [4,5,6].

Bat species can adapt to the urban environment with varying effectiveness, see reviews: [2,7]. Like other animals, they can be categorized regarding the extent of their adaptation. Urban exploiters frequently use anthropogenic shelters and are food generalists; urban avoiders usually are associated with old tree stands [8]. Fast flying bats commuting in open or edge spaces inhabit urban areas more often and accustom to urban conditions more easily than those that naturally use cluttered spaces, e.g., forests [2,9].

In general, when considering the activity and species diversity of bats in the city-outskirts gradient, increased values of these indices are observed in rural, mainly forested areas [10,11,12]. Therefore, many researchers underline a need for maintaining the proper condition of urban greenery, such as city parks. They serve as a para-natural structure of the land providing shelters, food and migration routes, and they can mitigate the negative impact of urbanization on bats [13,14,15].

To date, data regarding the use of urban habitats by bats from Central Europe are scarce [11,16]. Most studies were conducted in Australia, e.g., [15,17], North America, e.g., [18], South America e.g., [8,14], Mediterranean region, e.g., [19,20], or United Kingdom, e.g., [21]. These regions differ significantly from Poland in terms of climatic conditions and bat species composition. Additionally, most publications do not consider the impact of phenology on urban space used by bats in the annual cycle. To complement the knowledge, in our study, we aimed to evaluate the species composition and yearly activity of bats in Cracow’s Planty Park during the growing seasons in 2016 and 2017, regarding the influence of weather and the moon phase.

## 2. Materials and Methods

### 2.1. Study Area

The research was conducted in 2016 and 2017 in the Planty Park in Cracow, the city situated in southern Poland (19°56′ E 50°04′ N). It is the second-largest Polish city, with over 750,000 inhabitants. The climate is warm and temperate, with an average annual temperature 9.1 °C and annual precipitation 738 mm (Statistical Bulletin of the City of Cracow, 2017). Cracow is located within the land basin, which blocks the airflow and hence reinforces the urban heat island effect [22].

Municipal green areas cover nearly 17% of the city area, mostly within suburbs. Urban parks constitute only 5.2% of the city area. Planty is one of 47 parks in Cracow, located in the Old Town district. It covers 21.55 ha and is a ‘green belt’ surrounding the city’s very center, being one of the most representative green areas. The stand is dominated by old maples (*Acer*), lindens (*Tilia*) and horse chestnut (*Aesculus*). Within the park, the monumental remains of old city walls are situated. Planty is also adjacent to the Vistula river.

### 2.2. Ultrasonic Recordings and Sound Analysis

The field surveys were carried out over the growing seasons in 2016 and 2017. Within each of the seasons, 4 phenological periods were determined, corresponding to different activities of bats in the annual cycle: (a) spring (1 April–27 May): migrations, the formation of breeding colonies; (b) early summer (28 May–22 July): breeding and raising offspring; (c) late summer (23 July–16 September): dispersion of colonies, the start of migration and mating behaviors; (d) autumn (17 September–15 November): migrations to hibernacula, mating season. In each period once a week on clear evenings, eight bat call recording sessions were conducted during a walk through the entire park.

We used the ultrasound detector D240x (Pettersson Electronic, Uppsala, Sweden) in the Edirol R09 recorder set during the research. The detector worked in a heterodyne and time expansion system (memory size 1.7 s, TEx10). The recordings were made with a manual trigger, i.e., released by the operator while detecting bats’ calls and immediately loaded onto the recorder. Since the heterodyne system is narrowband with bandwidth of 8 kHz (e.g., if set on 40 kHz, only the sounds from of frequency between 36 to 44 kHz are detectable) the supporting detector Pettersson D230 with frequency division system scanning continuously the frequency range between 10 to 120 kHz, was also applied to allow manual recording. Time expansion mode allowed species recognition.

**Figure 1:**
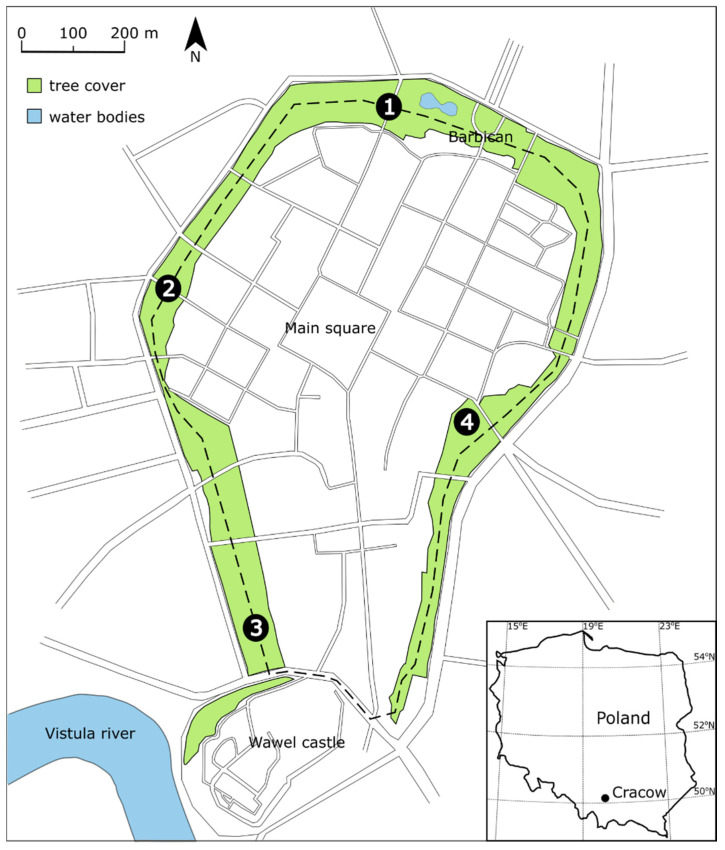
Planty Park in Cracow. The route followed by researchers during weekly recording sessions has been marked with a dashed line. Numbers refer to established checkpoints in which meteorological parameters were recorded.

Recordings started around sunset (±30 min) to capture the entire period of the highest diel activity of bats in the city over two seasons [11]. Each recording session started from one of the four designated characteristic checkpoints dividing the park into four segments (Figure 1). At each of these checkpoints, temperature, humidity and wind speed were measured with a Kestrel 4000 weather meter (Kestrel Meters, Boothwyn, PA, USA), and the cloud cover was visually assessed (arbitrary scale 0–100%). Each subsequent week, the recording session started from a different checkpoint than in the previous one; also, the direction of walking changed at least once during the phenological period to collect the recordings at every possible time on each segment. The moon phase was checked in the calendar in the evening of the recordings. The note form contained a map where numbers of recordings were marked according to the location certain recording was made. Usually, it took ca. 1.5 h to complete the session.

The recordings were subject to spectral analysis in the Kaleidoscope Viewer program (version 4.1.0, Wildlife Acoustics Inc., Maynard, MA, USA). We manually identified bat calls based on the parameters measured on a spectrogram, oscillogram and power spectrum plot: duration (dur), peak frequency (Fpeak), start frequency (SF), end frequency (EF) and bandwidth (BW, the difference between SF and EF). If the measured values fell within the range typical for species, the calls were classified to the species level; if the outcome was inconclusive, the calls were classified to the genus level or a group of species [23]. Social calls were additionally identified based on [24,25].

For individual species and groups of species, the number of bat passes was used as an overall activity indicator. Sequences of echolocation calls of similar characteristics in one-minute intervals were classified as a single flight [23]; adapted. The social and foraging activity, measured in the number of social call and number of feeding buzzes (short and frequent pulses at the end of echolocation sequences indicating the attack on the prey), was also noted.

### 2.3. Statistical Analysis

Bats richness and diversity was tested and determined based on Hill numbers [26,27,28], where:

− At q = 0, the abundances of individual species/taxa are not considered, so the value is simply the species/taxa richness of a given area;
− At q = 1, we obtain the Shannon diversity index, according to the Hill formula; very abundant and less abundant or rare species/taxa all have the same weight, i.e., the value obtained is the most neutral and indicates “true species diversity”;
− At q = 2, we obtain an index which is the reverse of Simpson’s index; Hill’s formula gives greater weight to more numerous and common species and less to rare species.

The two-way ANOVA was performed to test differences in bats’ activity (dependent variable) between the years and seasons (fixed variables). The data were transformed to meet the assumption of the normal distribution and homogeneity of variance (respectively, the Shapiro–Wilk test and the Levene test was applied). Afterwards, the post-hoc Tukey test was performed. Since the ranges of echolocation signals of species and their detectability by ultrasonic detectors differ significantly, we did not compare individual species and groups’ activities. The differences in bats’ overall activity between seasons were tested within groups of species assigned to ecological guilds, based on sonar characteristics and foraging ecology: (a) long-range echolocators (LRE) *Nyctalus*, *Eptesicus*, *Vespertilio*, (b) medium-range echolocators (MRE) *Pipistrellus* spp., *Hypsugo savii* and (c) short-range echolocators (SRE) *Myotis* spp. [23,29].

Because the bats pursue different life goals in the course of the year, the regression models were performed for the bats’ groups to identify the predictors explaining the bats’ activity within the seasons. The dependent (explained) variable was bats’ activity, and the explanatory predictors were factors measured during surveys: temperature, humidity, cloud cover, moon phase and wind speed. A two-step procedure was employed: We conducted the Spearman rank correlation and generated matrix plots for each factor and bats’ group activity (in analyzed season). It was to determine any relationship between bats’ activity and measured factors. Then, we constructed the regression models for factors that had the statistically significant relationship with bats activity and that relationship could be assumed to be linear. For the latter reason some data had to be transformed. If only one factor was taken into the model, the linear regression was performed, if two, backward stepwise regression. All performed models met the assumption of a normal distribution of residuals, absence of autocorrelation of factors and autocorrelation of residuals according to the Durbin Watson statistic values.

The richness and diversity analyses were performed in PAST 4.04 [30], other analyses were conducted in STATISTICA 13.2 for windows (Statsoft©, Inc., Tulsa, OK, USA).

## 3. Results

### 3.1. Bats’ Activity

Five species from the long-range echolocators group (LRE, i.e., *Nyctalus*, *Eptesicus* and *Vespertilio*), and five species from the medium-range echolocators group (MRE, i.e., *Pipistrellus* and *Hypsugo*) were found in the study area. Table 1 presents the number of recorded sequences depending on the type of bats activity.

### 3.2. Richness and Diversity between Seasons

**Figure 2:**
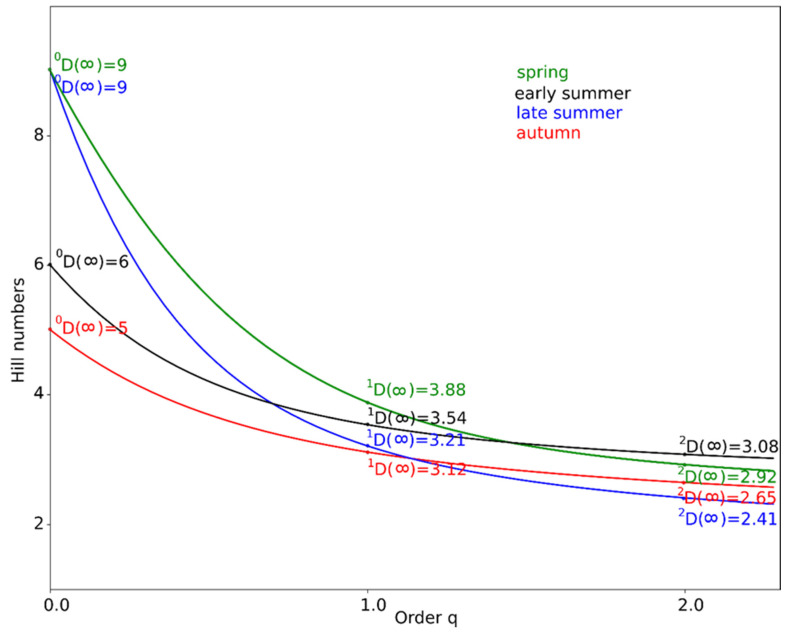
Diversity profile curve plotting Hill numbers for spring, early summer, late summer and autumn for bats (details in text).

Analyses of richness and diversity were conducted for the taxons presented in Table 1, with modifications: records of *H. savii/P. kuhlii* and unidentified species of *Pipistrellus/Hypsugo* were not taken into the analysis, as they cannot serve as an independent taxon. For the same reason, the records of *Nyctalus* sp. and unidentified specimens within LRE group were also removed from the analyses. The *Myotis* spp. group was included in the analysis as a single taxon. With those assumptions, identified bats (species, pair of species or taxon level) constitute 72% of all registered. We recorded the highest number of species in spring and late summer (^0^D = 9). The lowest number of species occurred in autumn (^0^D = 5). We found no great differences in diversity between seasons. The values of species richness and diversity for seasons are presented in Figure 2.

### 3.3. Seasonal Differences in the Bats’ Activity

**Figure 3:**
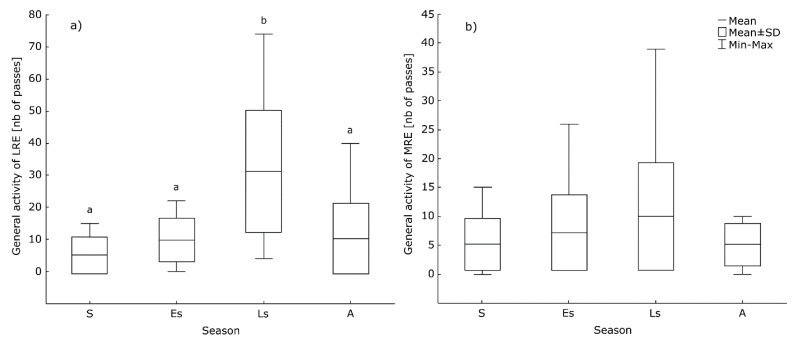
Seasonal differences in the activity of (**a**) long-range echolocators (LRE) and (**b**) medium-range echolocators (MRE). Abbreviated seasons’ names are as follows: S—spring, Es—early summer, Ls—late summer, A—autumn. Identical letters or lack of letters above boxplots indicate a lack of significant differences between seasons.

Two-way ANOVA revealed no significant differences in bats’ activity between the years of surveys (F = 0.36; *p* = 0.553 for long-range echolocators group, F = 0.0; *p* = 1 for medium-range echolocators group). Analysis showed significant differences between the seasons for long-range echolocators (F = 5.637; *p* < 0.0001); the greatest activity was found for late summer (Figure 3a). We found a similar trend of increased activity in the late summer in medium-range echolocators group, however, it was not statistically significant (F = 2.23; *p* = 0.09) (Figure 3b). The data concerning the short-range echolocators (*Myotis* spp.) were too scarce to conduct the analysis. Carrying out analyses for groups and not for species resulted from a large share of call sequences that could not be assigned to the species level.

### 3.4. Predictors of Bats’ Activity within the Seasons

The data for analyses were pooled together due to the lack of statistically significant differences in bats’ activity between years. Within all factors proposed as predictors of bats activity measured during each session, the matrix plots indicated potential predictors for each model. If only one factor was taken into the model, the linear regression was performed (model for the representation of the analyzed population: y = b_0_ + b_1_x_1_); if two—backward stepwise regression (y = b_0_ + b_1_x_1_ + b_2_x_2_) where y—bats’ activity; b_0_—intercept; b_1_, b_2_—regression coefficients; x_1_, x_2_—predictors. Table 2 contains the parameters’ values for each regression equation model, coefficients of regression (β) which allow to compare each factor’s relative contribution to the prediction of the bats’ activity, and the corrected (adjusted) coefficient of regression (R^2^ adjusted) informing about the explained variance. In one factor regression, β is equal to the correlation coefficient (R).

For instance, for long-range echolocators group in the spring model, where the matrix plots allow to include two predictors (temperature and cloud cover), the stepwise backward regression was performed. Only the temperature was statistically significant. The final equation of the model of regression was y = −1.774 + 0.213x. The value of the coefficient of regression (β) equaled the correlation coefficient (R), here: 0.846. The value of the corrected coefficient of regression (R^2^ adjusted) equaled 0.696. Hence, the factor (temperature) explained 69.6% of bats’ activity variability, and the rest of the variability relies on other factors not included in the model (Table 2).

## 4. Discussion

### 4.1. Species Composition and Activity

In our study, we observed all types of bats’ activity: social activity measured in the number of social calls sequences, general activity measured in the number of bat passes and foraging activity measured in the number of feeding buzz sequences. The percentage of unidentified echolocation calls sequences results from overlapping ranges of different species calls’ parameters.

Social activity concerned mainly the common noctule *Nyctalus noctula*, which is also the most frequently recorded species in the long-range echolocators group (*Nyctalus*, *Eptesicus* and *Vespertilio* genera). It undertakes long-distance migrations to wintering sites; however, recently, researchers often observe the year-round use of cities, where bats use buildings as roosts [31,32]. Our research results indicate the noctule’s population in Cracow is at least partially sedentary because of its high activity in all seasons; moreover, social calls occurring throughout the year indicate the presence of roosts in the park—most likely tree hollows and holes in Old City walls. We also confirmed another species of the genus, the Leisler’s bat *Nyctalus leisleri*, within all the investigated phenological periods. Although in Ireland it is one of the most common bats frequently roosting in buildings [33,34], in Poland it is considered a rare and sensitive species; in European cities, it is found in relatively small numbers [11,19,35]. Our results may indicate an ongoing synurbization process in this species.

The serotine (*Eptesicus serotinus*) was the second most abundant species from long-range echolocators group. It frequently occurs in Europe—it was found in large numbers from Kharkiv [36], Brno [11] or Madrid [19]. The northern bat (*Eptesicus nilssoni*) is not typically perceived as a synurbic species, although it may use urban space for foraging [37,38,39]. The particoloured bat (*Vespertilio murinus*) is known from large cities, mainly in Central and Eastern Europe [35,36]; in Poland: [40]. In this study, it was identified mainly based on the mating social calls recorded during autumn; however, a large fraction of individuals possibly remained unrecognized due to overlapping parameters of the echolocation calls with parameters typical for other species in this group.

From the medium-range echolocators group (*Pipistrellus*, *Hypsugo*) we recorded all species from these genera observed in Poland. It is particularly interesting to note the presence of two species very rarely found in Poland: the Savi’s pipistrelle (*Hypsugo savii*) and the Kuhl’s pipistrelle (*Pipistrellus kuhlii*).

The first of these species has been found in Poland only once in 2013 in the Carpathians, and the circumstances of finding the individual suggested its passive transport from Slovakia [41]. In Europe, most of its new records are in urban areas [41], and urbanization processes may favor the expansion [42]. Several observations of this species in our research may suggest the beginning of regular settlement in Poland. It is worth emphasizing that the site in Cracow is currently the northernmost known record site (the latitude of 50°04′ N) of the Savi’s pipistrelle in Central and Eastern Europe.

The Kuhl’s pipistrelle *P. kuhlii* in Poland has been found incidentally in the Warsaw city and Zawiercie town [43,44]. In southern Europe, it is considered a highly synurbic species [6,20]. In recent years, its expansion to the northeast has been observed, probably related to climate warming [44,45]. The finding of *P. kuhlii* in Cracow in all seasons serves as an update on its range and allows to recognize this species as a regular resident, potentially even abundant in Poland—especially in cities. Perhaps due to competition, other pipistrelle species: *P. pipistrellus* and *P. pygmaeus* in Planty were rarely found, although in urban areas, they can occur in vast numbers [21,46]. Along with recording *P. kuhlii* in Poland, the problem with distinguishing it from *P. nathusii* emerged due to overlapping calls’ parameters. *P. nathusii* also often inhabits cities and forms sedentary populations [47]. In our study, the complex of two species of *P. kuhlii*/*P. nathusii* constitutes the largest fraction of recorded bat passes in the medium-range echolocators group.

We rarely recorded *Myotis* bats in our study, which is similar to other studies carried out in urban parks [16,19,20]. *Myotis* bats are prone to negative effects of urbanization; they are usually recorded in suburban areas [48,49], particularly in the vicinity of rivers [11].

Overall foraging activity of bats was very low (13 recorded sequences), suggesting that Planty Park is not a suitable foraging site for species occurring there. The park most likely constitutes a commuting route to the Vistula river, where feeding buzzes for all groups of species were frequently observed [50].

### 4.2. Richness and Diversity of Bats between the Seasons

In our study, the highest species richness was observed in spring and late summer. Higher species richness in these two periods seems to be related to the emergence of certain species in the Planty Park (e.g., *P. pipistrellus*, *P. nathusii*, *P. kuhlii* and *H. savii*) (Table 1, Figure 2). Presumably, they use the Planty Park as a migration corridor and stop-off site.

Our results indicate that the species diversity of urban bat assemblages between all discussed seasons is similar. A possible reason is that the structure and composition of bat assemblages may be underestimated due to difficulties with recognizing short-range echolocators species (especially *Myotis* spp.) and assessing their activity.

Our results may also be the effect of the urban heat island phenomenon, causing the mild winters. The emergence of the new wintering areas in cities for species such as *N. noctula* and *P. nathusii* was explained by either urban heat island or/and greater accessibility of suitable shelters [31,47]. It has been proven for birds that urbanization increased the probability of year-round residence of migratory species [51]. It should be considered in future research of urban bats’ ecology.

### 4.3. Seasonal Differences in Bats’ Activity

In the two most common groups of bats recorded at Planty (long-range echolocators and medium-range echolocators), we observed a clear peak of general activity in late summer. However, we obtained statistical significance only for the former group (Figure 3). An increase in bats’ activity (the number of recorded bat passes) may result from the increase in their numbers, caused by the influx of young individuals from local breeding colonies and migrants [52]. An analogous and similarly explained bat activity pattern (shown for *N. noctula* and *P. nathusii*, as common and therefore representative species) was recorded on agricultural land in Germany [53]. In Brno, an increase in the activity of *P. pipistrellus* was observed in August, but for *N. noctula* and *E. serotinus* in April and July, which was purportedly related to the increased biomass of insects in the study area at that time [11].

### 4.4. Predictors of Bats’ Activity within the Seasons

Temperature predicted the activity of both tested groups of bats in the spring—activity increased with increasing temperature. This relationship can be associated with a low mass of adipose tissue after the hibernation period leading to greater susceptibility to adverse effects of low temperatures, mitigated by torpor [54]. We also obtained a similar outcome for long-range echolocators in early summer, and for medium-range echolocators in autumn, which may result from a higher abundance of insects under these conditions.

For medium-range echolocators, we observed an interesting phenomenon in early and late summer, when activity decreased with increasing temperature. Other studies held outside urban areas showed opposite tendencies [55]. This phenomenon did not appear in the long-range echolocators group. During the summer, the temperature often exceeded 20–25 °C. *Pipistrellus* and *Hypsugo* are small-sized bats that fly at rather low heights, while *Nyctalus*, *Eptesicus* and *Vespertilio* are larger and fly at higher heights. For this reason, *Pipistrellus* and *Hypsugo* bats could have been more exposed to the effects of high temperatures due to the evening radiation of the heat accumulated during the day on the ground surface. Although it is believed that small animals tolerate higher temperatures better due to the high ratio of body surface area to its volume (according to Bergmann’s rule), rapid temperature fluctuations can be hazardous for them due to the risk of dehydration, also resulting from a large body surface [56]. In bats, this tendency may be even more pronounced due to the construction of their wings, increasing body surface and possibly contributing to water loss. This topic requires special attention when analyzing the effects of climate change on living organisms.

In long-range echolocators, the activity decreased with increasing cloudiness in autumn. This type of relationship has occurred in another study in *N. noctula* [53]; it is also known from the literature for *Pipistrellus* and some *Myotis* species [55]. This phenomenon has not yet been fully explained. Researchers postulate that in migratory species such as *Nyctalus* and *Vespertilio*, the cloudy sky may hinder migration by covering stars and the moon, which potentially act as landmarks for bats [57]. Additionally, dense clouds diffuse city light, brightening the sky in urban areas, which may increase the risk of predation, especially in high-flying and open-air species, such as *Nyctalus*, *Eptesicus* and *Vespertilio* bats [57].

## 5. Conclusions

This research is one of the few that analyzes bats’ activity in the city in an annual cycle. It complements the knowledge on the use of urban space by bats and the ranges of some Mediterranean species (*Pipistrellus kuhlii*, *Hypsugo savii*), confirming their expansion to the north. We showed that urban parks could be an element of a migration route and also a space for establishing colonies and mating sites for some species. As bats are strictly protected animals across the European Union, it is necessary to exercise particular caution in urban green management.

## Figures and Tables

**Table 1:** List of recorded species and groups of species within ecological guilds, with total numbers of bat passes (BP), feeding buzzes (FB) and social calls (SC).

Sonar Type/Taxon	BP	FB	SC
Long-range echolocators			
*N. noctula*	470	2	45
*N. leisleri*	54	0	1
*Nyctalus* sp.	57	0	2
*E. serotinus*	85	1	0
*E. nilssoni* ^S, Es, Ls^	16	0	0
*V. murinus* ^A^	1	0	5
Unidentified	222	1	2
TOTAL	905	4	55
Medium-range echolocators			
*P. kuhlii*	19	0	1
*P. nathusii* ^Ls^	3	0	3
*P. kuhlii/P.nathusii*	338	0	0
*P. pygmaeus* ^S, Ls^	4	0	0
*P. pipistrellus* ^S^	1	1	0
*H. savii* ^S, Ls^	5	0	0
*H. savii/P. kuhlii*	12	0	0
Unidentified	63	8	2
TOTAL	445	9	6
Short-range echolocators			
*Myotis* sp.	21	0	0
Overall bat activity	1371	13	61

Superscript letters refer to seasons in which species were recorded (S—spring, Es—early summer, Ls—late summer, A—autumn). Lack of any mark indicates species recorded in all of the seasons.

**Table 2:** Results of applied models of regression to determine the impact of weather predictors on an activity of long-range echolocators group (*Nyctalus*, *Eptesicus* and *Vespertilio* genera) and on medium-range echolocators group (*Pipistrellus* and *Hypsugo* genera) within each analyzed season (detailed description in text).

		Spring			Early Summer			Late Summer			Autumn		
		β	b	p	β	b	p	β	b	p	β	b	p
Long-range echolocators	Intercept	-	−1.774	0.005	-	−0.650	0.461	-	-	-	-	2.614	<0.001
	Temperature	0.846	0.213	0.00004	0.654	0.132	0.006	nq			ns		
	Cloud cover	ns			nq			nq			−0.598	−0.001	0.01
	Humidity	nq			nq			nq			nq		
	Moon phase	nq			nq			nq			nq		
	Wind speed	nq			nq			nq			nq		
	Regression	Stepwise backward			Linear			-			Linear		
	R^2^ adjusted	0.696			0.387			-			0.312		
Medium-range echolocators	Intercept	-	0.558	0.239	-	3.973	0.0002	-	3.691	0.00006	-	0.822	0.01
	Temperature	0.575	0.080	0.02	−0.564	−0.098	0.02	−0.554	−0.072	0.03	0.658	0.075	0.006
	Cloud cover	ns			nq			nq			ns		
	Humidity	nq			nq			nq			a		
	Moon phase	nq			nq			nq			nq		
	Wind speed	nq			nq			nq			nq		
	Regression	Linear			Linear			Linear			Stepwise backward		
	R^2^ adjusted	0.282			0.270			0.257			0.392		

ns—not significant; nq—not qualified to the model by matrix plots; a—removed from the model due to autocorrelation with other factors.

## Data Availability

The data presented in this study are available on request from the corresponding author. The data are not publicly available due to the large size of the files’ library.
